# Analysis of Gene Expression in Resynthesized *Brassica napus* Allopolyploids Using Arabidopsis 70mer Oligo Microarrays

**DOI:** 10.1371/journal.pone.0004760

**Published:** 2009-03-10

**Authors:** Robert T. Gaeta, Suk-Young Yoo, J. C. Pires, R. W. Doerge, Z. Jeffrey Chen, Thomas C. Osborn

**Affiliations:** 1 Department of Agronomy, University of Wisconsin, Madison, Wisconsin, United States of America; 2 Department of Statistics, Purdue University, West Lafayette, Indiana, United States of America; 3 Department of Soil and Crop Sciences, Texas A&M University, College Station, Texas, United States of America; University of Massachusetts Amherst, United States of America

## Abstract

**Background:**

Studies in resynthesized *Brassica napus* allopolyploids indicate that homoeologous chromosome exchanges in advanced generations (S_5∶6_) alter gene expression through the loss and doubling of homoeologous genes within the rearrangements. Rearrangements may also indirectly affect global gene expression if homoeologous copies of gene regulators within rearrangements have differential affects on the transcription of genes in networks.

**Methodology/Principal Findings:**

We utilized *Arabidopsis* 70mer oligonucleotide microarrays for exploring gene expression in three resynthesized *B. napus* lineages at the S_0∶1_ and S_5∶6_ generations as well as their diploid progenitors *B. rapa* and *B. oleracea*. Differential gene expression between the progenitors and additive (midparent) expression in the allopolyploids were tested. The S_5∶6_ lines differed in the number of genetic rearrangements, allowing us to test if the number of genes displaying nonadditive expression was related to the number of rearrangements. Estimates using per-gene and common variance ANOVA models indicated that 6–15% of 26,107 genes were differentially expressed between the progenitors. Individual allopolyploids showed nonadditive expression for 1.6–32% of all genes. Less than 0.3% of genes displayed nonadditive expression in all S_0∶1_ lines and 0.1–0.2% were nonadditive among all S_5∶6_ lines. Differentially expressed genes in the polyploids were over-represented by genes differential between the progenitors. The total number of differentially expressed genes was correlated with the number of genetic changes in S_5∶6_ lines under the common variance model; however, there was no relationship using a per-gene variance model, and many genes showed nonadditive expression in S_0∶1_ lines.

**Conclusions/Significance:**

Few genes reproducibly demonstrated nonadditive expression among lineages, suggesting few changes resulted from a general response to polyploidization. Furthermore, our microarray analysis did not provide strong evidence that homoeologous rearrangements were a determinant of genome-wide nonadditive gene expression. In light of the inherent limitations of the *Arabidopsis* microarray to measure gene expression in polyploid *Brassicas*, further studies are warranted.

## Introduction

Polyploidy is a pervasive phenomenon in flowering plants that has contributed to their evolution and phenotypic variation [1]–[8]. Efforts to elucidate the mechanisms leading to novel variation in polyploids have included studies in polyploid *Arabidopsis*, *Brassica*, *Triticum*, *Gossypium*, *Nicotiana*, *Senecio*, *Spartina*, *Tragopogon*, and Triticale [9]–[20]. Some of these studies have been conducted on recent or resynthesized allopolyploids with known parents, and a theme has emerged: genetic, epigenetic, and transcriptional changes are all common observations in newly formed polyploids (reviewed in [6], [21]–[23]). Biased expression of homoeologous transcripts has been observed in *Gossypium* polyploids [11], and the qualitative loss and duplication of homoeologous genes has been detected in *Brassica napus* [18]. Similarly, loss of progenitor cDNA amplified fragment length polymorphisms (cDNA-AFLPs) has been reported in studies of polyploid *Arabidopsis*, *Triticum*, *Brassica*, and *Tragopogon* [17]–[20]. In some cases evidence for epigenetic or genetic mechanisms that lead to the observed changes in gene expression have also been reported. Transcriptional changes are likely to be a critical component of polyploid evolution because they can contribute directly to novel phenotypes; however, little is known about how polyploidy causes transcriptional changes or the impact of these changes on phenotypes.

Microarray technologies allow for genome-wide analysis of large numbers of genes in parallel, and have been adopted for studies of polyploidization in resynthesized plant polyploids. Wang et al. (2006) analyzed expression in resynthesized *Arabidopsis* allopolyploids and reported that 3.1% of nearly 26,000 genes reproducibly showed nonadditive expression in two independent lineages (under the intersection of genes significant under both per-gene and common variance models); however, up to 38% of the transcriptome showed expression changes within lines [24]. This study also included an analysis that compared diploid and autotetraploid lines of *A. thaliana*, which found that few changes in gene expression resulted from autopolyploidization (∼0.3%). In resynthesized *Senecio cambrensis* hybrids microarray analysis detected significant changes in gene expression [25], [26]. Polyploidization of these hybrids appeared to stabilize the expression of many genes in a manner consistent with natural *S. cambrensis* polyploids. A study of gene expression in a *Solanum phureja* autopolyploid series (1X–4X) concluded that nearly 10% of genes displayed changes among ploidy levels, most of which occurred at the monoploid level [27].

Doubled haploid (DH) *B. rapa* and *B. oleracea* lines were previously used as parents in generating a population of resynthesized *B. napus* allopolyploids that were analyzed for genetic, epigenetic, gene expression, and phenotypic changes at both the S_0∶1_ and S_5∶6_ generations (S_0_ derived S_1_ plants were bulked to represent each S_0_ line, and S_5_ derived S_6_ plants were bulked to represent each S_5_ line) [18], [28]. Homoeologous chromosomal exchanges detected in S_5∶6_ lines were associated with the loss of specific parental gene transcripts and an increase of the other parental homoeologous transcript. The number of rearrangements was correlated with the overall level of phenotypic variation generated among the S_5∶6_ polyploid lines, suggesting that loss and doubling of homoeologous genes was an important cause for novel phenotypic variance in this population. Although loss and doubling of homoeologous genes affects the composition of homoeologous transcripts, it may not affect the overall expression of homoeologous sets of genes in rearranged chromosomes. However, homoeologous exchanges could indirectly alter genome-wide gene expression detectable by microarrays if homoeologous copies of gene regulators contained in rearrangements have differential affects on the transcription of genes in networks.

**Figure 1:**
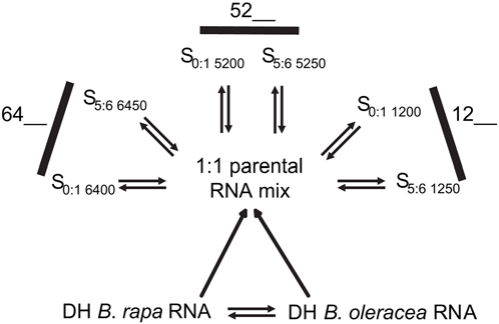
Microarray Experimental Design for Analysis of *Brassica* Diploid and Resynthesized Polyploid Gene Expression. Doubled haploid (DH) inbred lines of *B. rapa* (line IMB218) and *B. oleracea* (TO1000) were compared, and a 1 to 1 mix of diploid RNA was used as a reference sample for comparisons with three resynthesized *B. napus* lines at both the S_0∶1_ and S_5∶6_ generations. Opposing arrows represent two dye-swap comparisons on each of two biological replicates of each line (8 microarray hybridizations per comparison).

To test the relationship between homoeologous rearrangements and quantitative changes in genome-wide gene expression in resynthesized *B. napus*, we used the *Arabidopsis* 26K spotted 70mer oligonucleotide microarray (accession number GPL7536) to compare gene expression levels between the diploid progenitors, and among three independently resynthesized allopolyploid lines at both the S_0∶1_ and S_5∶6_ generation (Figure 1). The three lineages were chosen on the basis of differing numbers of genetic changes detected at the S_5∶6_ generation [18]. This allowed us to test whether the total number of genes displaying nonadditive (i.e., deviated from midparent value) expression was related to the number of chromosome rearrangements in the lines. Differential expression was tested using two linear (analysis of variance; ANOVA) models. The first model assumed a common variance for all genes, while the second linear model relied on limited biological replication to estimate the individual gene variation (i.e., per-gene variance). Our previous studies using this array platform determined that the sources of variation were similar in hybridization experiments with both *Brassica* and *Arabidopsis* species [29], [30], and that verifiable changes in candidate gene expression could be detected in natural *B. napus* polyploids following infection with *Sclerotinia* [31]. Although this microarray is unable to distinguish between homoeologs or paralogs, we expected it could detect overall expression changes in sets of homoeologous or paralogous genes, which might occur through the differential affects of homoeologous gene regulators contained in rearranged chromosomes. In addition to testing the main hypotheses, the biological functions of differentially expressed genes were investigated. For the purposes of this study differentially expressed orthologous genes were classified according to *Arabidopsis* gene annotations. Finally, we compared our findings with results from other microarray studies in resynthesized allopolyploids.

## Results

### Summary of Genes Differentially Expressed between Diploid Progenitors: *B. rapa* and *B. oleracea*

As explained, the total number of differentially expressed genes between the diploid progenitors was determined using two linear (analysis of variance; ANOVA) models (Table 1; Dataset S1). Under the per-gene variance model, 3980 (15% of 26,107) *Arabidopsis* genes represented on the microarray demonstrated significant differential expression between the diploid progenitors, and approximately equal numbers of up and down regulated genes were detected in the parents (one sample test of equal proportions; *X* ^2^ = 0.93; *P* = 0.33). Under the common variance model 1627 (6% of 26,107) genes showed significant differential expression, and the proportion down regulated in *B. rapa* (54%) was significantly different from the proportion down regulated in *B. oleracea* (46%) (*Χ* ^2^ = 10.39, *P* = 0.001). Only 1% of all genes were differentially expressed under both models, and the proportion of genes down regulated in *B. rapa* (61%) was different from the proportion down regulated in *B. oleracea* (39%) (*X* ^2^ = 13.1, *P*<0.001).

**Table 1:** Summary of Differentially Expressed Genes.

Comparison	Total No. of Genetic Changes^a^	Total No. DEG^b^ per-gene variance	Total No. DEG^b^ common variance	Total No. DEG^b^ intersection of both models
*rapa vs oleracea*	N.A.	3980	1627	284
Down in *rapa*		1959	879	173
Down in *oleracea*		2021	748	111
Mix vs 6400	0	8230 (20%)^c^	1230 (26%)^c^	70 (17%)^d^
Up		4511	572	30
Down		3719	658	40
Mix vs 5200	0	545 (30%)^c^	952 (22%)^c^	12 (42%)^d^
Up		188	434	5
Down		357	518	7
Mix vs 1200	0	424 (46%)^c^	833 (26%)^c^	11 (55%)^d^
Up		153	440	5
Down		271	393	6
S_0∶1_ DEG^a^ Overlap	N.A.	79 (52%)^c^	69 (43%)^c^	3 (100%)^d^
Up		31	28	0
Down		48	27	3
Mix vs 6450	2	1139 (37%)^c^	760 (26%)^c^	12 (33%)^d^
Up		536	373	4
Down		603	387	8
Mix vs 5250	16	809 (29%)^c^	810 (26%)^c^	6 (33%)^d^
Up		581	417	4
Down		228	393	2
Mix vs 1250	28	1002 (30%)^c^	856 (21%)^c^	17 (47%)^d^
Up		573	405	6
Down		429	451	11
S_5∶6_ DEG^a^ Overlap	N.A.	52 (56%)^c^	36 (47%)^c^	1 (100%)
Up		28	11	0
Down		20	9	1

Note: 6400, 5200, and 1200 are S_0∶1_ generation lines and 6450, 5250, and 1250 are corresponding S_5∶6_ lines.

a Genetic changes included total number of RFLP and SSR marker fragment losses (Gaeta et al., 2007).

b DEG = Statistically significant differentially expressed genes using FDR (0.05) under per-gene or common gene variance models.

c % differentially expressed genes that were also differential between the diploid progenitors under the given variance model. The proportion of genes that demonstrated nonadditive expression in the allopolyploids that were also differential between the parents was significantly greater than would be expected by random chance. We performed Chi-square tests of equal proportions using R statistical software, *P*<0.00125 (alpha = 0.01/8; significance levels adjusted by a Bonferrroni correction for the eight comparisons conducted under each variance model).

d % differentially expressed genes that were also differential between the diploid progenitors under the given variance model. The proportion of genes that demonstrated nonadditive expression in the allopolyploids that were also differential between the parents was significantly greater than would be expected by random chance. Tests involving these proportions required a Fisher Exact Test using R statistical software because of low cell counts, *P*<0.00125.

### Summary of Nonadditive Gene Expression among Resynthesized *B. napus* Allopolyploids

**Figure 2:**
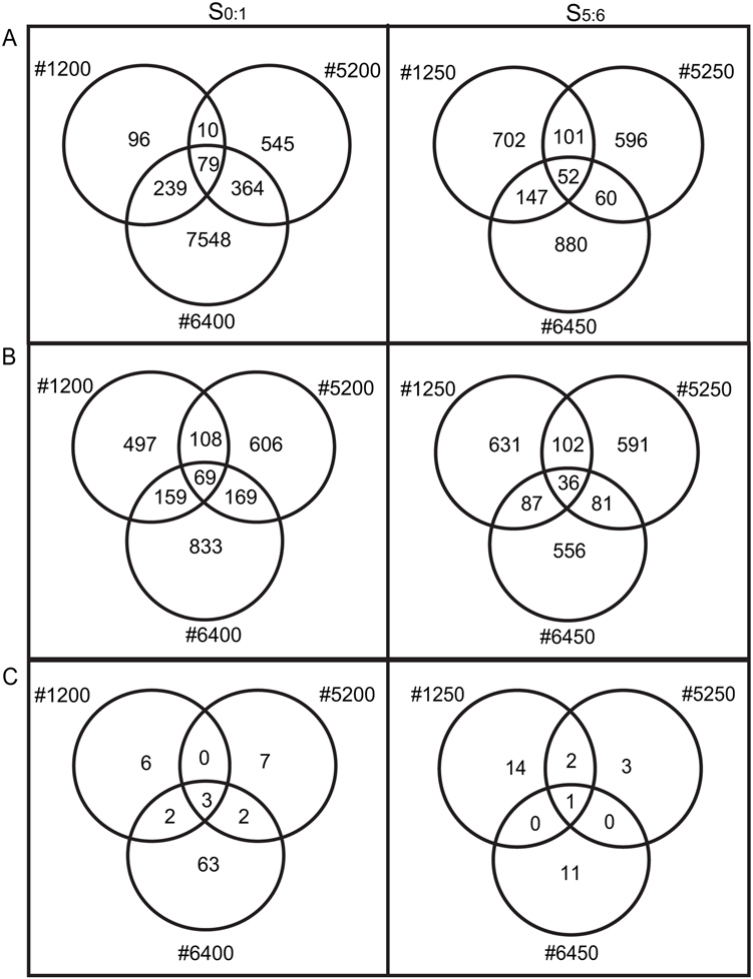
*Venn* Diagrams Summarizing the Number of Differentially Expressed Genes Detected in Each Allopolyploid at the S_0∶1_ and S_5∶6_ Generations. The number of differentially expressed genes detected in the S_0∶1_ and S_5∶6_ generations (left and right panels, respectively) under the A) per-gene variance ANOVA model, B) common variance ANOVA model, and C) intersection of the per-gene and common variance models.

Under the per-gene ANOVA model, 1.6 to 32% (ave. 11.7%) of all genes displayed nonadditive expression among S_0∶1_ allopolyploids, and 3.1 to 4.4% (ave. 3.7%) demonstrated nonadditive expression among S_5∶6_ allopolyploids (Table 1; Datasets S2, S3, S4, S5, S6, S7, S8). Significantly more genes showed up regulation relative to the midparent expression value in comparisons with lines 6400, 5250, and 1250, and significantly more genes were down regulated in comparisons with lines 5200 and 1200 (one sample *X* ^2^ tests of equal proportions with Bonferroni correction; *P*<0.00625; Table 1). Only 79 genes (0.3%) and 52 genes (0.2%) reproducibly demonstrated nonadditive expression in all three lines at the S_0∶1_ and S_5∶6_ generations, respectively. No significant bias in the number of up or down regulated genes was observed among the 79 and 52 genes that reproducibly changed in three lines at the S_0∶1_ and S_5∶6_ generations, respectively. The genes differentially expressed in the allopolyploid comparisons were significantly overrepresented by those differentially expressed between the diploid progenitors (Table 1, see footnote c), and were equally represented by genes up or down regulated in the progenitors (data not shown). The numbers of differentially expressed genes shared among the three lines at the S_0∶1_ and S_5∶6_ generations are displayed in Figure 2A. Sixteen genes were differentially expressed in all three lines in both generations under the per-gene ANOVA model, nine of which have no known function (Table S1).

When the individual variance assumption was relaxed and all genes were assumed to have the same variance (i.e., common variance ANOVA model), 3.2 to 4.7% (ave. 3.8%) of genes demonstrated nonadditive expression among S_0∶1_ allopolyploids, and 2.9 to 3.3% (ave. 3.1%) showed significant differences among S_5∶6_ allopolyploids (Table 1; Datasets S2, S3, S4, S5, S6, S7, S8). There was no significant difference in the number of up and down-regulated genes detected using the common variance model (one sample *X* ^2^ tests of equal proportions with Bonferroni correction; Table 1). Only 69 genes (0.3%) and 36 genes (0.1%) demonstrated nonadditive expression in all three lines at the S_0∶1_ and S_5∶6_ generations, respectively. The genes differentially expressed in allopolyploids were significantly overrepresented by genes differentially expressed between the progenitors (Table 1, see footnotes c and d), and were equally represented by those up or down regulated in both progenitors (data not shown). The numbers of differentially expressed genes shared among the three lines at the S_0∶1_ and S_5∶6_ generations are displayed in Figure 2B. Seven genes demonstrated differential expression in all lines in both generations under the common variance model, three of which have no known function (Table S1).

Only a few genes were significant in the intersection of the per-gene and common variance ANOVA model results (Table 1; Figure 2C). This result is due to the magnitude of an expression change relative to the variation of the gene. Namely, those genes in common between the two analyses (common and per-gene variance assumption) generally demonstrated very large changes in expression levels while having small variances. Because of the relatively small number of biological replicates, the per-gene variance model was restricted in its ability to estimate the variance of each gene while the common variance assumption model was dominated by the genes with large changes that typically had small variances. As such, the two statistical models detected rather distinct subsets of differentially expressed genes. Approximately equal numbers of up and down regulated genes were detected under the intersection of both models (Table 1), and these were significantly overrepresented by genes differentially expressed among the diploid parents (Table 1, footnotes c and d). The intersection of the two statistical models revealed a single gene with unknown function that was down regulated in all lines in both generations (Table 1 and Table S1).

**Figure 3:**
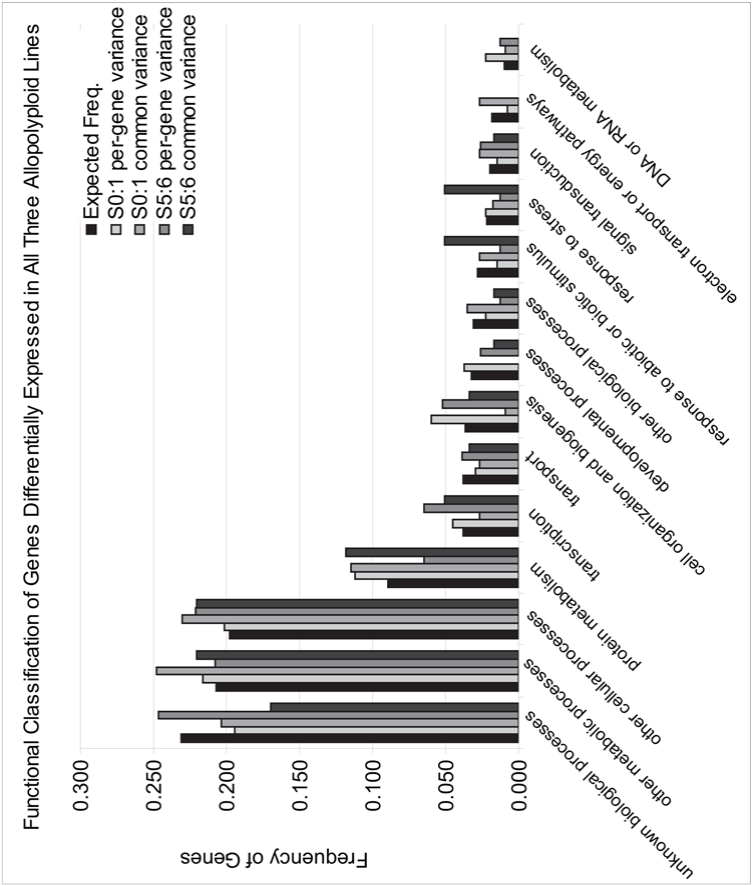
Biological Functions of Genes Demonstrating Nonadditive Expression in all Three Lines. Genes that reproducibly displayed nonadditive expression in all allopolyploid lines at the S_0∶1_ or S_5∶6_ generations under the per-gene and common-variance models were characterized according to biological function (http://www.arabidopsis.org/tools/bulk/go/index.jsp). Expected frequencies in each category were estimated based on annotation of the entire *Arabidopsis* genome.

Genes that demonstrated differential expression under either the per-gene or common gene variance ANOVA models in all three lines at the S_0∶1_ or S_5∶6_ generations were classified by function (Figure 3; Dataset S8). The percentage of genes in each category under each model for both S_0∶1_ and S_5∶6_ generations did not significantly differ from expected ratios (based on GO classification of all *Arabidopsis* genes; *P*-values were ≥0.51 for each model). Therefore, no functional category of genes was over or under represented in lists of differentially expressed genes.

Previous analyses of these allopolyploids detected no genetic changes in the S_0∶1_ generation (lines 6400, 5200, and 1200); however, in the S_5∶6_ generation line 6450 had 2 genetic changes (0.5% of all markers), line 5250 had 16 genetic changes (3.7% of all markers), and 1250 had 28 genetic changes (6.6% of all markers) (Table 1; [18], [28]). In the S_5∶6_ generation, a positive correlation between the number of differentially expressed genes and genetic changes (sum of RFLP and SSR DNA marker losses per line; [18]) was detected under the common variance ANOVA model (Pearson correlation = 0.99, *P* = 0.0129; Spearman rank correlation = 1, *P*<0.0001).

### Confirmation of Differential Gene Expression by Quantitative RT-PCR

Microarray results were confirmed using real time quantitative RT-PCR for 14 genes that were differentially expressed (Table 2; See Methods for how genes were chosen). The expression of each gene in IMB218 was calculated relative to TO1000 expression levels, and the expression of each gene in the six allopolyploids was calculated relative to the 1 to 1 parent mix sample. A total of 98 comparisons (14 genes and 7 comparisons per gene) were tested and compared with microarray results (Dataset S9). Thirty-eight comparisons were expected to exhibit no significant difference in expression based on microarray analysis, and of these 24 (63%) were confirmed as having no difference in expression, 14 (37%) demonstrated a significant difference when the array predicted no difference (false negative) (FDR, 0.05). Sixty comparisons were predicted to exhibit differential expression based on microarray analysis, and of these 29 (48%) were confirmed to be differentially expressed and the direction of change was congruent (*P*<0.05), 8 (13%) were differentially expressed, but the direction of change was opposite to that predicted by the microarray analysis (*P*<0.05), and 23 (38%) were not significantly different (false positives). Of these sixty predicted expression changes, 25 were significant under both models, 32 were significant under the per- gene variance model only, and 3 were significant under the common gene variance model only. There was no significant difference between the rate of confirmable changes predicted by the per-gene model, the common gene variance model, or overlap of both models (*P* = 0.85). In summary, of 98 total comparisons the results of 53 (54%) were confirmed, 8 (8%) demonstrated opposite expression, 14 (14%) false negatives were detected, and 23 (23%) false positives were detected. Among the 14 genes, the individual confirmation rates ranged from 100% (7/7 comparisons confirmed) to 14% (1/7 comparisons confirmed), with most analyses confirming ∼4/7 comparisons (Dataset S9).

**Table 2:** Summary of Genes Analyzed by Quantitative RT-PCR.

Target no.	Oligo ID	*Arabidopsis*/*Brassica* 1 Loci	Biological Function in *Arabidopsis*	Quantitative RT-PCR Primers
Tub.	A018922_01	At5g44340	protein polymerization	F-GTCTGTGACATTGCACCAAAG
		DY017618		R-GTCCATGCCTTCTCCTGTGT
2	A002166_01	At1g71695	peroxidase/stress response	F-TAGTTGCACTTTCAGGTGGC
		CD845448		R-GTGTGTTGCTCGAGTTAGCG
4	A002902_01	At1g76920	ubiquiton protein ligase	F-AGAGAGCTTGGAGTGGGAGG
		CD818935		R-AGCTTCCCCATCCTCTTAGC
5(5-1)	A003095_01	At1g17750	transmembrane protein kinase	F-AAGCAGCTACGAGGATGACG
		DU832841		R-CACCACATCTCTCATGGACG
12	A006045_01	At2g18710	SECY protein translocase	F-CAGTACAATGTGATTTGATGGTAAT
		AM386952		R-GCAAGAAAGGTTCAAGCTGAG
19	A008305_01	At2g42840	protodermal factor (PDF1)	F-GCTCTCTACCGTGAAGGCAC
		CA991909		R-TATGGGCCTGCTTAGTTGCT
21^2^	A008716_01	At2g17620	Cyclin-dependent	F-TCCTGTCAATTTCCCCGTAG
		DU102054	protein kinase	R-ATGGTTACAGGCAATGGAGC
22	A008717_01	At2g26580	YABBY-like	F-TGCACCAATCTGTGGTCTGT
		CD830187	transcription factor	R-AATTTTGGTGTGGCCTCTTG
32	A014325_01	At4g13040	AP2-domain transcription factor	F-GTTGGTTCCCTTCCACACAT
		CN730283		R-GGCAAGCAGCCATTAAAGTT
37	A015606_01	At5g64330	blue-light response	F-TAGCCCATCGTCACAACTCC
		BH420489		R-TCAGAACGCGAAGATGAGAGT
41	A018670_01	At5g57010	calmodulin-binding protein	F-TGGAAAGAATTGGAATTGGC
		BZ484870		R-ACCTTTGCTGCTTTTGTTCC
45	A021226_01	At3g48630	unknown	F-GTGTGCCTCAACAAGCAAGATTG
		CN731576		R-TAAGAACCGCCAAGTGTGTGTCA
48^2^	A021566_01	AT5g26130	pathogenesis-related protein	F-AGATTCGTACATTCCGGTGG
		AF370026		R-ATGCATGTGTTCGAAGCGTA
53	A022180_01	At3g49550	unknown	F-GAGTCCGGTTAGTTTGCAGC
		H663133		R-ATCTCCCATGGTCACCTCTG
65	A025930_01	At4g12300	cytochrome P450	F-TGAACGCTTCCTTAAGCTCC
		BZ613137		R-CGAAGCTGCGGTTAGATTGT

1 Targets orthologous to 70mer oligo sequences were identified by blast search of the *Brassica* DNA database (http://www.arabidopsis.org/wublast/index2.jsp).

2 Expression was only observed in the TO1000 parent by quantitative RT-PCR.

**Figure 4:**
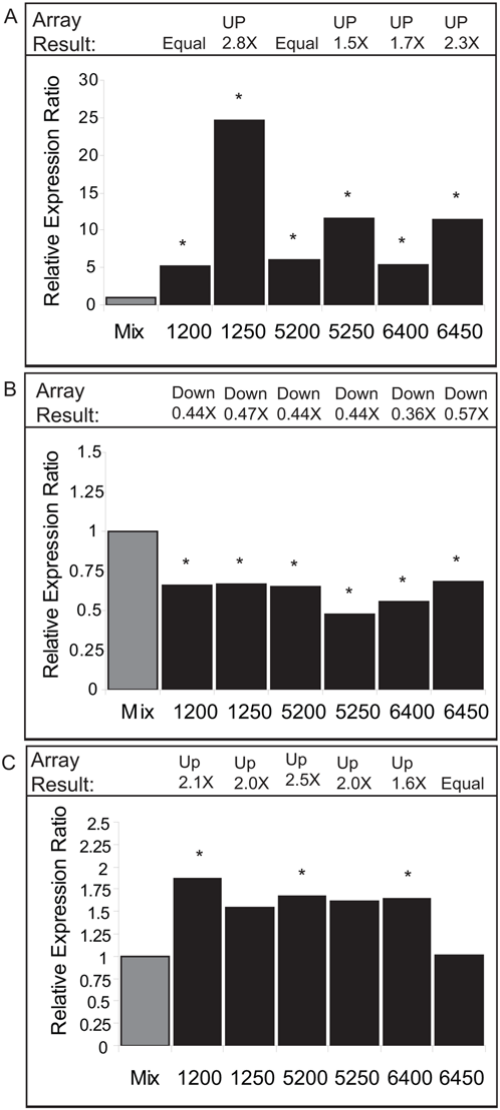
Quantitative RT-PCR Confirmation of Three Genes Displaying Nonadditive Expression Patterns among Allopolyploids. Expression ratios (y-axis) were estimated from the difference between normalized CT values measured in the reference sample (1 to 1 mix of parental RNA) and allopolyploid samples (2^[difference in normalized CT values]^). Expression ratios detected by microarray analysis are indicated at the top of each bar graph (represents the fold change difference between reference and allopolyploid samples). Asterisks indicate that statistically significant differences in normalized LS-mean CT values were detected between reference and allopolyploid samples (See Methods; * = *P*<0.05). A) Up-regulation of gene At5g26130 (the stress-response gene in *Arabidopsis* similar to *Brassica* sequence AF370026) was observed in all allopolyploids. B) Down-regulation of At2g18710 (a SECY protein translocase in *Arabidopsis* similar to *Brassica* cDNA AM386952) in all allopolyploids. C) Up-regulation of At1g17750 (an LRR protein kinase in *Arabidopsis* similar to *Brassica* sequence DU832841) was predicted in five of six allopolyploids and a trend in expression similar to the expected was observed; however the difference detected by quantitative RT-PCR was only statistically significant for lines 1200, 5200, and 6400.

Several genes demonstrated up or down regulation in most or all lines that were analyzed, some of which were verified by quantitative RT-PCR (Figure 4). A pathogenesis related gene (*Brassica* EST AF370026 is similar to *Arabidopsis* accession no. At5g26130) showed the greatest deviation from additive expression levels among the allopolyploid lines by quantitative RT-PCR (Figure 4A), and expression exceeded high-parent (TO1000) levels by ≥2 fold in all lines (not shown). The microarray analysis indicated that four of six lines were up regulated at this locus, and quantitative RT-PCR detected up regulation in all lines. Expression was nearly absent in the *B. rapa* parent IMB218 for this gene (>6000 fold less than TO1000 levels; not shown); consequently transcripts measured in the allopolyploids may represent only those derived from TO1000 (data not shown). PCR analysis of parental DNA samples detected a faint band in IMB218 of the same molecular weight to that observed for TO1000 (data not shown); however direct sequencing of the PCR reaction did not indicate similarity with AF370026, suggesting genetic divergence among the progenitors. Another gene (*Brassica* cDNA AM386952 which is similar to At2g18710) functions as a SECY protein translocase in *Arabidopsis*, and demonstrated down-regulation in all allopolyploid lines as the microarray predicted (Figure 4B). A third gene (*Brassica* clone DU832841 which is similar to At1g17750) functions as a LRR protein kinase in *Arabidopsis* and was predicted by array analysis to be up-regulated in five of six lines. For this gene, quantitative RT-PCR confirmed the trend in expression for the lines, but only three lines demonstrated a statistically significant difference (Figure 4C). In several other cases the trend in expression detected by quantitative RT-PCR coincided with changes predicated by the microarray, but the differences were not statistically significant (as in Figure 4C).

### Identification of Genes Displaying Differential Regulation in *Arabidopsis suecica-*like and *Brassica napus*-like Allopolyploids

Wang et al., 2006 reported that 820 genes displayed nonadditive expression in two independent *A. suecica* allopolyploid lines (allo733 and allo738) under the intersection of per-gene and common variance ANOVA models. We compared these results to our results (both statistical models) that displayed nonadditive expression in all three *B. napus* allopolyploids at either the S_0∶1_ or S_5∶6_ generation. A total of eight genes were identified that were detected in both microarray studies (Table 3). Some of these genes showed the same pattern of expression across the two species (up or down regulated relative to midparent), while others displayed opposite patterns of expression. Five of these eight genes (63%) were transcription factors, three of which were identified as significantly differentially expressed under the common variance analysis of all three S_0∶1_ *B. napus* allopolyploids.

**Table 3:** Genes Differentially expressed in *Arabidopsis* 1 and *Brassica* Allopolyploids.

Oligo ID	*Arabidopsis* locus	Biological Function	Expression Changes in Allopolyploids^2^
A012110_01	AT3G09360 (RNA pol II)	transcription regulation	dn *A.s.* (∼0.54) per/common
			up *B.n.* (1.3–1.4) per-gene (S_5∶6_)
A021798_01	AT2G29480 (ATGSTU2)	glutathione transferase	dn *A.s.* (0.42–0.57) per/common
			dn *B.n.* (0.53–0.57) per-gene (S_0∶1_)
A023454_01	AT2G26150 (HSFA2)	heat stress response transcription factor	dn *A.s.* (0.45–0.60) per/common
			up *B.n.* (2.7–7.2) common (S_5∶6_)
A000929_01	AT1G80840 (WRKY40)	stress response transcription factor	dn *A.s.* (0.07–0.08) per/common
			up/dn *B.n.* (0.20–3.4) common (S_0∶1_)
A008067_01	AT2G42360	zinc finger (C3HC4-type RING finger) family protein	dn *A.s.* (0.39–0.47) per/common
			up *B.n.* (3.0–5.1) common (S_0∶1_)
A013098_01	AT1G76880	trihelix DNA-binding transcription factor	up *A.s.* (1.7–2.7) per/common
			dn *B.n.* (0.30–0.39) common (S_0∶1_)
A019376_01	AT5G14760	L-aspartate oxidase	dn *A.s.* (0.26–0.53) per/common
		NAD biosynthesis	dn *B.n.* (0.22–0.41) common (S_0∶1_)
A021641_01	AT2G05310	unknown	up *A.s.* (2.0–2.8) per/common
			up *B.n.* (2.5–4.2) common (S_0∶1_)

1 Based on data from Wang et al., 2005.

2 The range in fold change values observed in two *A. suecica*-like (*A.s.*) allopolyploids (Wang et al., 2006) and three *B. napus*-like (*B.n.*) allopolyploids for this gene; up = up-regulation relative to midparent value; dn = down regulation relative to midparent value. The *Arabidopsis* data is based on the intersection of per-gene and common variance estimates from two lines (allo733 and allo738) and *Brassica* data was based on either the per-gene or common variance estimates for all three lines at either the S_0∶1_ or S_5∶6_ generation (as indicated).

## Discussion

Previous studies on a population of resynthesized *B. napus* lineages reported genetic changes in all lines at the S_5∶6_ generation, many of which resulted from homoeologous chromosome rearrangements [18]. In that study, total genetic changes (measured as DNA fragment losses) were positively correlated with total cDNA-AFLP changes (measured as fragment losses) and phenotypic variability [18]. The qualitative nature of this previous study did not allow for a genome-wide quantitative assessment of gene expression, and did not test whether gene expression levels differed from the midparent value (additivity). Furthermore, it did not address whether or not homoeologous exchanges might also lead to global changes in the expression of genes, possibly through altering the dosage of homoeologous trans-acting regulatory factors. In this study we attempted to address these questions using an *Arabidopsis* 70mer oligonucleotide microarray. This microarray was limited by a design that was based on sequences from a different species, and an inability to distinguish between related transcripts. Consequently, the changes in gene expression reported could have resulted from the differential hybridization of one or more members of a group of related gene transcripts. Despite limitations, some of our observations are consistent with data from other polyploid studies.

### *Arabidopsis* Microarrays Detected No Effect of Chromosome Rearrangements on Genome-wide Nonadditive Gene Expression in Resynthesized *Brassica napus*

In this study, we selected lines with differing numbers of genetic changes in the S_5∶6_ generation and tested whether they were related to the number of genes demonstrating nonadditive expression. In the S_5∶6_ generation, a positive correlation between genetic changes and DEGs under the common variance ANOVA model was detected. However, since related transcripts could not be discerned, we could not determine if this relationship was due to changes in the expression of homoeologous genes within rearrangements or whether it reflects changes in the expression of genes regulated by genes within the rearrangements. Furthermore, we could not determine whether changes in gene expression resulted from changes in the expression of a single gene or multiple related genes. A correlation was not detected under the per-gene variance model. This came as no surprise given that few changes in gene expression were significant under both statistical models, yet makes it difficult to draw biological conclusions. The limited numbers of biological replicates provide one potential reason that the two models selected different subsets of differentially expressed genes. Therefore, variance in our analysis may have been too high to detect a stronger effect of rearrangements. Differentially expressed genes were also readily detected in the S_0∶1_ generation. Our previous data suggested that some chromosomal rearrangements observed in the S_5∶6_ generation initiated from homoeologous recombination (reciprocal exchange) in early generations [18]; however, we could not determine whether these exchanges impacted gene expression in the S_0∶1_ since they were undetectable by the genetic analysis employed. Overall, these data do not provide strong evidence that genetic changes were a major determinant of genome-wide nonadditive gene expression.

Previous data suggested that genome rearrangements in these resynthesized *B. napus* lines contributed to qualitative changes in the expression of homoeologous (or parental-allele-specific) transcripts; however, it is unknown whether these changes lead to deviations from midparent expression (quantitative additivity). In one example, Gaeta et al. (2007) presented expression data for a gene (*pW225; At4g32251*) in which the loss of a homoeologous transcript corresponded with an increased dosage of the other, such that total expression did not appear to change. Lines 1250 and 5250 both contained homoeologous nonreciprocal transpositions (HNRTs) that altered homoeologous expression of *pW225* transcripts, yet both lines exhibited midparent expression of transcripts orthologous to *Arabidopsis* gene (At4g32551) in our microarray analysis. Line 6450 on the other hand, which was qualitatively additive for parental *pW225* DNA and transcripts, demonstrated a significant deviation from midparent expression levels in the microarray analysis. These data suggest that changes in the expression of homoeologous genes may not affect the total expression level of the combined homoeologs, and thus may not necessarily lead to deviations from the midparent value. Alternatively, this may indicate that for some genes the *Arabidopsis* microarray could not detect changes in gene expression caused by homoeologous rearrangements. It remains to be determined if nonadditive expression detected for other genes in our microarray analysis might be explained by specific homoeologous rearrangements in the lines, since the microarray platform we employed was unable to distinguish between related transcripts.

### Few Genes Demonstrated Nonadditive Expression Among Independently Resynthesized *Brassica napus* Lineages

Few studies of resynthesized allopolyploids have analyzed multiple independent lines, making it difficult to draw general conclusions. Genes that reproducibly displayed expression changes among all three allopolyploids were probably not due to genetic changes, because there were no genetic changes shared in common by all three S_5∶6_ allopolyploids. These changes in gene expression may represent a general response to polyploidization in resynthesized *B. napus* that is unrelated to lineage-specific genome rearrangements. We found that approximately 0.3% of 26,107 *Arabidopsis* genes demonstrated nonadditive expression in all three allopolyploid lines at the S_0∶1_ generation, and 0.1 to 0.2% of all genes were differential in all three lines at the S_5∶6_ generation. Therefore, most genes did not reproducibly show nonadditive expression among independent lines, regardless of the statistical model employed. The results are similar to findings in resynthesized *Arabidopsis* allopolyploids in which ∼3.1% of genes displayed differential expression in two independent lines [24], illustrating the importance of analyzing multiple independent polyploids before making generalizations about the effects of polyploidization. Thus, results from these two related polyploid species suggest that many changes in gene expression within independently resynthesized lines were random, and many genes show additive expression. Several studies in polyploid species have reported the general observation that many genes assayed by microarray tend to be expressed at midparent levels [25], [26], [27], [32].

In our previous study of a population of nearly 50 resynthesized *B. napus* allopolyploids we found that hotspots in the *B. napus* genome were more likely to undergo homoeologous rearrangement than others, suggesting that many qualitative changes in homoeologous gene expression may be directed rather than random [18]. Together with our current analysis, these data suggest that both random and non-random changes in gene expression occur in resynthesized *B. napus*. The combined effects of both random and non-random changes on gene expression could contribute to novel variation during polyploid evolution. Estimates using different ANOVA models suggest that independent lineages may display changes in up to 32% of all genes in *Brassica* and up to 38% in *Arabidopsis*, suggesting most variation in gene expression is lineage specific in both of these species. In *Brassica*, we observed roughly similar frequencies of up and down regulated genes across allopolyploid comparisons (see Figure 2) while in *Arabidopsis* allotetraploids differentially expressed genes were more often down-regulated [24]. In both of these studies, it was unknown whether hybridization or polyploidization per se was responsible for the nonadditive expression observed, since there were no diploid hybrids available for comparison.

We found that between 6% (common variance ANOVA model) and 15% (per-gene variance ANOVA model) of all genes displayed differential expression between the diploid progenitors (*B. rapa* line IMB218 and *B. oleracea* line TO1000). The genes significant under both the common gene variance ANOVA model and the intersection of both models were mostly down regulated in *B. rapa* relative to *B. oleracea*, although this bias was relatively small (Table 1). Wang et al. (2006) reported that between 17% (common variance) and 43% (per-gene variance) of genes displayed differential expression between *A. thaliana* and *A. arenosa* diploid progenitors, more of which showed lower expression in *A. thaliana* relative to *A. arenosa*. In both of these polyploid systems, the genes that displayed nonadditive expression in the allopolyploids were overrepresented by genes differentially expressed in the diploid progenitors. Consequently, the variation in gene expression observed in these two species may have been somewhat dependent upon expression variation between the progenitors. Similar observations have been made at the protein level in resynthesized *B. napus*, in which newly formed polyploids reproducibly demonstrated non-additivity for 25 to 38% of >1600 proteins surveyed in roots and stems, and nonadditive proteins were overrepresented by those with differences between the parents [33]. Further studies that include multiple independent polyploids from each of several sets of distinct parents could address the question of whether or not expression divergence among progenitors contributes to the magnitude of nonadditive expression in resynthesized allopolyploids.

The two statistical models that were employed provided largely disparate lists of statistically significant genes in all comparisons. High variance due to the cross-species nature of our polyploid microarray analysis, combined with the fact that we had a limited number of biological replications may have contributed to this. However, most of the biological observations held up under either model and have been made in other allopolyploid studies. These data indicate that the use of different statistical models in the face of increased variation, as well as different microarray platforms [34], can affect the results of microarray analyses of polyploid transcriptomes. For these reasons, we summarized data derived from both statistical analyses, and focused on genes that were differentially expressed among multiple independent lines.

### Confirmation of Gene Expression Changes in Resynthesized *B. napus*

The confirmation rate we observed in our study was similar to those reported in microarray analyses of other polyploid species. We were able to confirm approximately 54% of the results (including both confirmation of equal and unequal expression) for 14 genes. We detected both false negatives (14%) and false positives (23%), and significant changes in the opposite direction (8%). Some results might not have been confirmed due to the different sources of error across experimental platforms (i.e., microarray vs quantitative RT-PCR). Poole et al. (2007) was able to confirm approximately 62% of the changes observed in a microarray analysis, and this was similar to other reports in wheat [35]. In microarray analyses in *Senecio* allopolyploids, quantitative RT-PCR confirmation rates were also approximately 65% [25], [26].

The accuracy of the *Arabidopsis* microarray we used for analysis of *Brassica* polyploids could have been affected by sequence divergence. Similarity in sequence between diploid *Brassica* species and *Arabidopsis* has been estimated to be approximately 87% [36]. As a consequence, estimates of gene expression may have been less accurate for genes that have significantly diverged in sequence across the two species. Hudson et al. (2007) employed statistical methods for filtering out such features, leading to increased accuracy in a heterologous microarray analysis of *B. napus* [37]. We attempted to partially mitigate this shortcoming by using a lower hybridization temperature (55°C), although this may have contributed to increased cross-hybridization and false positive or negative results. However, our previous studies demonstrated similar sources of variation in experiments with *Arabidopsis* and *B. oleracea*, and >95% of *Arabidopsis* oligos hybridized well to *Brassica* cDNA [29], [30]. Recently this microarray was used in an analysis of gene expression in response to *Sclerotinia* infection in *B. napus*, and the authors reported verifiable differences in expression of genes linked to resistance QTL [31]. Thus, in the absence of a comprehensive homoeolog-specific *Brassica* array, *Arabidopsis* microarrays continue to be an important tool for genome-wide exploration of gene expression in *Brassica* species. The caveat is that such analyses will require more extensive validation experiments depending upon the goals of the study, especially if biological conclusions are to be drawn regarding the consequences of any particular change in gene expression.

The genomes of diploid *Brassica* species are triplicated relative to *Arabidopsis* [38], and allopolyploids may express six or more distinct transcripts that correspond to a single copy gene in *Arabidopsis*. The platform we utilized in the current study was unable to distinguish among related transcripts (homoeologs or paralogs), but should have been capable of measuring changes in sets (or subsets) of related transcripts. In some instances it is possible that microarray features differed in their specificities for duplicate gene transcripts. For example, confirmation rates could be affected where RT-PCR primers and microarray oligos had differing specificities for related *Brassica* transcripts [39], [40]. A multiplatform analysis of gene expression in wheat polyploids highlighted the issue of how specificity among different platforms may affect microarray results [34]. Recently, a cotton microarray was designed with probes capable of distinguishing homoeologous transcripts [32], [41]; however, there has been little progress in the development of genome-wide homoeolog-specific microarrays in other plant polyploid systems. These studies exemplify the continued need for techniques that can discriminate between homoeologs or parent specific transcripts in allopolyploids (i.e., RT-PCR SSCP, CAPS, cDNA-AFLPs, and homoeolog-specific microarrays; see [30], [41], [42]. Because of the variety of methods that are available for analyzing gene expression in polyploids, conclusions based upon changes in gene expression must be taken in the context of how they were measured, and the limitations of the detection system.

### No Particular Biological Process was Prone to Differential Regulation in Resynthesized *B. napus* Allopolyploids

To date, microarray studies of polyploidization in newly resynthesized *Senecio* and *Arabidopsis* allopolyploids have found few if any common sets of differentially expressed genes [24]–[26]. The biological functions of genes that reproducibly displayed differential expression in all *B. napus* lineages were not over or under-represented by any specific functional class of gene. This result is in accordance with observations at the protein level in root and stem tissues of resynthesized *B. napus* allopolyploids [33], [43]. However, we cannot rule out that changes in the expression of parent-specific transcripts or duplicates may have occurred more or less frequently in specific functional categories, since our microarray could not measure this. Microarray analyses in *Senecio* allopolyploids and hybrids similarly found that no particular functional category of genes was overly affected; however, the authors did mention a slight overrepresentation of stress and defense genes [25], [26]. In *A. suecica*-like allopolyploids, hormone regulating and stress-related genes were the most overrepresented [24]. In our study we also detected changes in stress-responsive genes using the common variance model (although the overrepresentation was not statistically significant), indicating that up regulation of this class of gene may be a general phenomenon in newly resynthesized allopolyploids. When gene lists were compared between microarray analyses of *A. suecica* [24] and *B. napus* allopolyploids, the few genes in common were mostly transcription factors; however, the number of genes overlapping from the two experiments was so few it would be expected by random chance. Further studies would be needed to verify the potential importance of these genes.

### Conclusion

Given the inherent detection limitations of *Arabidopsis* microarrays for measuring the expression of duplicated transcripts in *Brassica* polyploids and the limited number of available biological replications, it is likely that our analysis was hindered by both biological and technical variation. This is evidenced by the lack of concordance between the two ANOVA models and by the relatively low confirmation rate achieved with quantitative RT-PCR. The two ANOVA models appeared to detect distinct subsets of genes as significant, and were only in agreement for genes demonstrating large fold changes and low variances. However, several new observations are consistent with previous studies of other resynthesized allopolyploids and warrant further investigation. Few genes reproducibly displayed nonadditive gene expression among three independently derived resynthesized *B. napus* lineages, suggesting that most of the changes observed within independently resynthesized *B. napus* lineages were lineage-specific, and thus mostly random. Overall, most genes generally showed additive expression. Genes that demonstrated non-midparent expression were overrepresented by genes differential in the progenitors. This could suggest that divergence in progenitor gene expression might correlate with the nonadditive expression in allopolyploids. While we observed a strong correlation between genetic changes and homoeologous gene expression in our previous work [18], we found little evidence that homoeologous genetic changes contributed to the overall number of genes displaying changes in expression. Further work is needed to determine the causes of these quantitative transcriptional changes and whether they contribute to phenotypic divergence in newly formed polyploids.

## Materials and Methods

### Microarray Experimental Design

We employed a dye-swap experimental design that included two biological and two technical replications (Figure 1; [29], [42]). Seven total comparisons were conducted: Gene expression in the diploid parents (*B. rapa* line IMB218 and *B. oleracea* line TO1000) was compared. A reference sample was created by mixing parental mRNA in a 1 to 1 ratio, and was compared to six polyploids (independently resynthesized S_0∶1_ lines 1200, 5200, and 6400, and their corresponding S_5∶6_ lines 1250, 5250, and 6450). Four dye-swap comparisons (2 for each biological replicate, involving a total of 8 microarray slides) were performed for each comparison, for a total of 56 hybridizations in the study.

### Plant Materials and RNA Extraction

Seed was sown in four-inch pots in Metro Mix soil. Two biological replicates were planted as separate blocks in an environmentally controlled growth chamber (Percival Scientific, Perry Iowa). Plants were watered daily and fertilized every other day as needed with dilute (1 tblsp/20 liters) Peters Professional Peat Lite Special 20-10-20. Temperature was maintained at 21°C and lighting was maintained at ∼258 and 280 micromoles/m^2^/s^−1^ in each replicate growth chamber, respectively, for 16 hrs each day. The two biological replicates of the parental genotypes were composed of pooled leaf tissue from 40 plants, and were arranged in flats of 10 plants. The two biological replicates for each polyploid genotype were composed of bulked leaf tissue from 10 plants (S_1_ and S_6_ plants from each line were bulked to represent the S_0_ and S_5_, respectively), and the 10 plants were grown in a single flat. Flat locations within each replicate were randomized weekly. All plants were harvested at the same developmental stage, when the third and fourth true leaves were outstretched from the meristem. Plants were harvested at the same time of day (11:00am to 12:00pm CST). Leaves two, three, and four from individual plants were bulked (as described above) comprising a given line replicate, and were flash frozen and homogenized in liquid N_2_ and stored at −80°C. Total RNA was extracted from each biological replicate using Tri-Reagent (Molecular Research Center Inc., Cincinnati, OH) according to manufacturer protocols, and was quantified using a NanoDrop ND-1000 (Wilmington, DE). Messenger-RNA was purified from total RNA using the Invitrogen FastTrack Micro mRNA Isolation Kit according to manufacturer protocols (Carlsbad, CA). The quality of extracted RNAs was confirmed by 1% agarose electrophoresis and 260/280 ratios.

### Preparation of Microarray Slides

A total of 27,648 *Arabidopsis* 70-mer oligo nucleotides (representing 26,107 *Arabidopsis* genes) were spotted onto >100 Super Amine microarray slides (ArrayIt, Sunnyvale, CA) using the OmniGrid Accent microarrayer (GeneMachines, San Carlos, CA) according to protocols described by Wang et al., 2005. Gene names, GenBank accession numbers, and 70mer sequences of the oligos can be found at http://www.operon.com/arrays/omad.php.

### RNA Labeling and Microarray Hybridization

For labeling mRNA, 500 ng of mRNA (in 15 µl) was combined with 1 µl of oligo (dT) (2 µg/µl), and 1 µl of random nonamer (2 µg/µl, Gene Link, Hawthorne, NY) in a 1.5 ml microcentrifuge tube on ice. Reactions were mixed and incubated at 65°C for 5 minutes. Samples were placed at room temperature for 10 minutes and briefly centrifuged. Six microliters of reverse-transcriptase buffer (5×), 3 µl of DTT (0.1 M), 1 µl of dNTP (10 mM dATP, dTTP, dGTP, 2.5 mM dCTP), 1.5 µl of Cy5- or Cy3-dCTP, and 1 µl of Superscript II Reverse Transcriptase (Amersham Biosciences, Piscataway, NJ; Invitrogen, Carlsbad, CA) were added to the reactions and mixed in a total volume of 30 µl. Reverse transcription reactions were incubated at 42°C for 2 hours under dark conditions. Three microliters of 2.5 M NaOH was added to each reaction, samples were mixed, and incubated at 37°C for 15 minutes in the dark. Fifteen microliters of 2 M HEPES was added and mixed. Labeled samples were then purified using the Qiagen PCR purification kit (Valencia, CA) and eluted in 30 µl of Buffer EB. For any given hybridization-comparison, reciprocally labeled samples (one Cy3 labeled, the other Cy5 labeled) were mixed and heated at 95°C for 2 minutes. Then 12 µl of 20× SSC, 2 µl of 10% SDS, and 7.5 µl of 10% BSA were added for a total hybridization volume of ∼75 µl. Hybridization to microarray slides and washing steps were performed as described by Wang et. al., 2005, except that hybridization was conducted at 55°C. Microarrays were scanned using the GenePix 4000B scanner (Axon Instruments, Inc., Union City, CA) and GPR files were generated for data analysis according to protocols described in [39].

### Microarray Data Analysis

The raw data were background corrected by subtracting the background median from the foreground median intensity for both red and green intensities; negative results were set to 1. A transformation was performed on background-corrected intensities by taking the natural logarithm. MA plots [44] were employed to investigate dye effects. Data were normalized using a robust local regression (loess function). Consistency and density plots were also used to investigate data quality. To identify differentially expressed genes between any two samples, two Analysis of Variance (ANOVA) models were used: The first model used a common variance assumption and the second model used per-gene variance assumption.

Specifically, the common variance ANOVA model employed is:
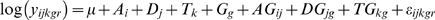
where 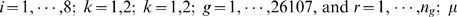 is the grand mean, and A, D, T and G are the array, dye, treatment and gene effects, respectively. Moreover, AG, DG and TG are the interactions between array and gene, dye and gene, and treatment and gene respectively. 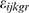 are error terms which are independent random variable form a normal distribution with a mean 0 and variance 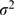. Using the common variance ANOVA model differential expression was tested using the following:
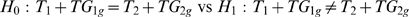



As mentioned a per-gene variance ANOVA model was also employed:
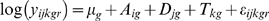
where μ_g_, A, D, T is the average gene intensity, array, dye and treatment effects for gene g respectively. 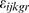 are error terms which are independent random variable form a normal distribution with a mean 0 and variance 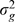. Using the per-gene variance assumption model, differential expression was tested using
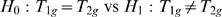
To accommodate the multiple testing issues that arise from testing differential expression of 26,107 genes for differential expression, Benjamini-Hochberg's FDR was employed to control the significance level at 0.05 (Benjamini and Hochberg 1995).

Among 26,107 genes, some genes were replicated 6, 48, 49, or 382 times on an array. Genes with large number of replication have more degree of freedom so they have more statistical power when testing for differential expression. To eliminate the replication imbalance and to put all genes on the same replication level, genes with replicates on the array were averaged and the average was considered as one feature for the analysis. It is necessary to point out that the statistical model that is based on a common gene variance assumption has more power simply because it assumes that all genes in the genome have the same variation; which is unlikely to be true across nearly 26,000 genes. The statistical model that is based on the per-gene variance assumption represents a more biologically realistic model since it analyzes each gene uniquely, yet is limited by the number of biological replicates in this study. In this study we summarize results that are based on independent analyses using these two models, as well as the intersection of results from both models. All raw data has been deposited in the public database Gene Expression Omnibus (GEO) under the following accession number: GSE13431.

### Identifying Biological Functions of Differentially Expressed Genes

Using tools on the TAIR website (www.arabidopsis.org/index.jsp) we categorized the differentially expressed genes according biological function. Expected frequencies for each category were calculated based on the entire database of annotated *Arabidopsis* genes.

### Selection of Genes and Primer Design for Confirmation Analysis by RT-PCR

For Real Time Quantitative RT-PCR, we initially selected 65 genes with various putative functions from lists of genes that demonstrated nonadditive expression under both ANOVA models for at least one comparison. From this list, we further selected genes showing differential expression in multiple comparisons, and tried to be sure at least one comparison showed equal expression in the array analysis in order to estimate false negatives. The 70mer oligo sequences corresponding to these genes (http://www.operon.com/arrays/omad.php) were used to identify orthologous *Brassica* sequences with WU-BLAST2 (http://www.arabidopsis.org/wublast/index2.jsp). We eliminated genes for which the 70mer feature on the microarray did not demonstrate homology to any known *Brassica* sequence. Primers were designed from *Brassica* sequences to target regions homologous to the *Arabidopsis* 70mer sequence. When possible, the consensus nucleotides between *B. rapa* and *B. oleracea* were used to target primers to conserved nucleotides. The primers amplified 100–200 bp cDNA products whose specificity was verified by direct sequencing of DNA and cDNA products from diploid parents and melting curve analysis. Primers were tested by real time PCR on a dilution series of cDNA (1 to 2, 1 to 4, 1 to 8, 1 to 16, 1 to 32, 1 to 64) derived from a mix of first strand cDNAs from the diploid parents TO1000 and IMB218 in triplicate, and primer efficiencies were calculated using REST-384© version 2 software (http://rest.gene-quantification.info/). All primers used for PCR analysis had comparable amplification efficiencies (1.9–2.1) and generated single, specific PCR products. Of the remaining genes that met the above selection criteria, we chose 14 at random for RT PCR analysis (Table 2).

### cDNA Synthesis for RT-PCR Experiments

The same total RNA samples used for microarray analysis were DNase treated with Ambion (Austin, TX) Turbo DNA-*free*™ DNase and quantified using a Nanodrop ND-1000 spectrophotometer (Wilmington, DE). For cDNA synthesis, 5 µg of DNase-treated total RNA was reverse transcribed with oligo d(T) primers using the Invitrogen Super Script II First Strand cDNA synthesis kit according to manufacturer protocols (Carlsbad, CA). Parallel control reactions (RT−) were also conducted on all samples of RNA. RT+ and RT− samples were screened with 12 of the 14 genes used for Real Time Quantitative RT-PCR. In a few instances negligible DNA contamination was detected (RT− samples reached the threshold of detection ∼8.8–15.4 CT values later than corresponding RT+ samples). RT+ and RT− samples were additionally screened with 14 primer sets designed from *Arabidopsis* gene annotations and no DNA contamination was detected by standard RT-PCR (not shown).

### Real Time Quantitative RT-PCR and Data Analysis

Real time quantitative RT-PCR was carried out using the DNA Engine Opticon 2 System (Bio-Rad; Hercules, CA). Reactions were set up by combining 10 µl of 2× Reaction Mix (New England Biolabs; Ipswich, MA) with 1.5 µl of 1 to 20 dilute cDNA templates, 1 µl of forward and 1 µl of reverse primer (10 µM each), and 6.5 µl of ddH_2_0. For gene expression analyses, we analyzed the control gene beta-Tubulin and one target gene per run, and reactions were carried out on 2 biological and 2 technical replicates of each sample. Reactions were placed in the thermocycler under the following conditions: 95°C for 15 min; 39 cycles of 94°C for 20 sec, 57°C for 30 sec, and 72°C for 30 sec, sample read; 72°C for 10 min; and melting curve analysis. The global minimum was subtracted for baseline correction. The threshold line was adjusted to be above early cycle background fluorescence and fluorescent intensities detected in water controls at ≥35 cycles, and to intersect the fluorescence curves in the middle of the exponential phase. Occasionally fluorescence was detected in no- template controls in later cyles (>35 cycles), and melting curve and gel analysis indicated the source was primer dimers. Data on the threshold cycle (CT) at which the fluorescent intensity of each sample first increased above background levels was collected, and was normalized to beta Tubulin levels (which showed very little expression variation among the nine samples analyzed in this study). Relative expression was calculated between *B. rapa* and *B. oleracea* or between the reference sample (1 to 1 parent mix) and the six allopolyploid samples using PROC MIXED in SAS Version 9.1: This analysis assumed equal primer efficiencies and used Tubulin CT values to calculate baseline corrected CT values for each gene of interest [45]. Pair-wise contrasts were used to estimate the difference in baseline adjusted CTs (difference of LS means) between reference and unknown samples. Since 1 CT = ∼2 fold change, these values were used to estimate relative fold change expression ratios between samples. To determine if an assumption of equal primer efficiencies was appropriate, efficiency adjusted relative expression ratios were calculated using REST-384© version 2 software. The expression ratios calculated using mixed model analysis in SAS correlated well with the efficiency-adjusted ratios calculated using REST-384 software (Spearman rank correlation  = 0.98, *P*<0.0001). We used SAS Version 9.1 to test whether differences in baseline corrected LS mean CT values from pair-wise contrasted samples were statistically significant [45]. We tested seven comparisons for each gene (14 genes and 7 comparisons per gene = 98 total comparisons). FDR was employed to control the significance level at 0.05 across 98 comparisons (Benjamini and Hochberg 1995).

## Supporting Information

Table S1 Biological Functions of Genes Displaying Nonadditive Expression in All Three Allopolyploid Lines in Both Generations. Supplemental Table summarizing genes that were differentially expressed in all three allopolyploids in both generations (S0 and S5) analyzed
(0.07 MB DOC)

Dataset S1 Summary of statistically significant genes that were differentially expressed between the diploid progenitors B. rapa and B. oleracea
(2.28 MB XLS)

Dataset S2 Summary of statistically significant genes that were nonadditively expressed in allopolyploid line 1200 relative to the 1∶1 parent mix.
(0.37 MB XLS)

Dataset S3 Summary of statistically significant genes that were nonadditively expressed in allopolyploid line 1250 relative to the 1∶1 parent mix.
(0.64 MB XLS)

Dataset S4 Summary of statistically significant genes that were nonadditively expressed in allopolyploid line 5200 relative to the 1∶1 parent mix.
(0.40 MB XLS)

Dataset S5 Summary of statistically significant genes that were nonadditively expressed in allopolyploid line 5250 relative to the 1∶1 parent mix.
(0.42 MB XLS)

Dataset S6 Summary of statistically significant genes that were nonadditively expressed in allopolyploid line 6400 relative to the 1∶1 parent mix.
(2.36 MB XLS)

Dataset S7 Summary of statistically significant genes that were nonadditively expressed in allopolyploid line 6450 relative to the 1∶1 parent mix.
(0.50 MB XLS)

Dataset S8 Summary of statistically significant genes that were nonadditively expressed in all S0 allopolyploid lines relative to the 1∶1 parent mix, all S5 allopolyploid lines relative to the 1∶1 parent mix, and all allopolyploid plants (S0 and S5) relative to the 1∶1 parent mix.
(0.04 MB XLS)

Dataset S9 Summary of Real Time RT-PCR confirmation analysis. This data set summarizes all microarray and RT-PCR expession changes and P-values for the 98 comparisons tested by quantitative RT-PCR in our microarray confirmation analysis.
(0.07 MB XLS)
