# Supplementary material for: Analysis of Gene Expression in Resynthesized Brassica napus Allopolyploids Using Arabidopsis 70mer Oligo Microarrays
Source: PLoS One. 2009 Mar 10;4(3):e4760. doi: 10.1371/journal.pone.0004760 (PMC2651575; doi:10.1371/journal.pone.0004760)
Supplement: Table S1 — Biological Functions of Genes Displaying Nonadditive Expression in All Three Allopolyploid Lines in Both Generations. Supplemental Table summarizing genes that were differentially expressed in all three allopolyploids in both generations (S0 and S5) analyzed (0.07 MB DOC) [file pone.0004760.s001.doc]

Table S1: Biological Functions of Genes Displaying Nonadditive Expression in All Three Allopolyploid Lines in Both Generations.

| **Oligo ID** | ***Arabidopsis* Locus** | **Biological Function/Process** | **(F.C.)^1^ in Allopolyploids** | ***P*-value^2^** |
| --- | --- | --- | --- | --- |
| A001812_01 | At1g69480 | unknown | up (1.2-1.4) | per-gene variance  *P* ≤ 0.000589 |
| A002874_01 | At1g15060 | unknown | up (1.5-2.2) | per-gene variance  *P* ≤ 0.000912 |
| A005137_01 | At4g27670 | response to stress/ abiotic or biotic stimulus | up (1.1-1.4) | per-gene variance  *P* ≤ 0.000563 |
| A006045_01^3^ | At2g18710^3^ | cell organization and biogenesis | dn (0.34-0.57) | per-gene variance  *P* ≤ 0.00000893 |
| A008358_01 | At2g04790 | unknown | dn (0.48-0.69) | per-gene variance  *P* ≤ 0.000499 |
| A011569_01 | At3g06130 | metal ion transport | dn (0.43-0.62) | per-gene variance  *P* ≤ 0.000386 |
| A011799_01 | At3g54750 | unknown | up (1.4-1.8) | per-gene variance  *P* ≤ 0.000855 |
| A012036_01 | At3g04280 | signal transduction | up (1.6-2.8) | per-gene variance  *P* ≤ 0.000906 |
| A014859_01 | At4g36600 | development | dn (0.63-0.83) | per-gene variance  *P* ≤ 0.001534 |
| A016175_01 | At5g65010 | asparagine biosynthesis | up (1.1-1.3) | per-gene variance  *P* ≤ 0.000686 |
| A019067_01 | At5g41640 | unknown | up (1.2-1.4) | per-gene variance  *P* ≤ 0.000615 |
| A019975_01 | At3g01060 | unknown | up (1.5-1.7) | per-gene variance  *P* ≤ 0.00128 |
| A020190_01 | At3g61240 | hydrolase, ATP dependent helicase | up (1.5-2.3) | per-gene variance  *P* ≤ 0.000502 |
| A021498_01 | At5g39800 | unknown | up (1.2-1.6) | per-gene variance  *P* ≤ 0.00158 |
| A022988_01 | At5g38550 | unknown | up (1.4-1.6) | per-gene variance  *P* ≤ 0.000639 |
| A023688_01 | At2g17750 | unknown | dn (0.17-0.30) | per/common  *P* ≤ 0.0000941 |
| A002879_01 | At1g15330 | unknown | up/dn (0.30-5.1) | common variance  *P* ≤ 0.000599 |
| A009001_01 | At3g45440 | kinase, protein metabolism | dn (0.08-0.31) | common variance  *P* ≤ 0.00000723 |
| A020590_01 | At5g48380 | kinase, protein metabolism | up/dn (0.14-2.5) | common variance  *P* ≤ 0.000368 |
| A022098_01 | At2g10350 | protein metabolism | up (2.7-25.4) | common variance  *P* ≤ 0.000771 |
| A023068_01 | At5g63230 | unknown | up/dn (0.12-3.9) | common variance  *P* ≤ 0.001201 |
| A024680_01 | At5g54270 | photosynthesis | dn (0.06-0.34) | common variance  *P* ≤ 0.000201 |

^1^The range in fold-change values observed among the six allopolyploids; up = up-regulation relative to mid-parent value; dn=down regulation relative to mid-parent value; up/dn = for this gene both and up and down regulation was observed among the polyploid lines.

^2^The largest *P*-value observed among the six allopolyploid comparisons for each gene is indicated as well as the variance model under which significance was determined.

^3^This gene was included in the Real Time RT-PCR confirmation study (See Table 3 and Figure 5B).
